# POORER SLEEP HEALTH MAY INCREASE THE RISK OF DEVELOPING PHYSICAL FRAILTY, PARTICULARLY AMONG WOMEN

**DOI:** 10.1093/geroni/igae098.0251

**Published:** 2024-12-31

**Authors:** Muhammad Thalil, Soomi Lee, David Almeida

**Affiliations:** The Pennsylvania State University, University Park, Pennsylvania, United States; The Pennsylvania State University, University Park, Pennsylvania, United States; Pennsylvania State University, University Park, Pennsylvania, United States

## Abstract

Longitudinal evidence on the association between sleep and physical frailty is limited, especially using a multidimensional measure of sleep health. In this study, we examined the prospective association between sleep health and physical frailty among U.S. older adults. We also examined how this association differs by age, sex and race. We used data from the National Health and Aging Trends Study (NHATS), with a sample of 929 older adults (65+) who completed a sleep module during 2013 and 2014. Self-reported items on the SATED model (i.e., satisfaction, daytime alertness, timing, efficiency, and duration) were used to create a sleep health composite and Fried’s frailty phenotype was used to assess frailty. Multiple multinomial logistic regression models adjusted for baseline frailty status, socio-demographics, and health correlates revealed that having poorer sleep health increased the risk of onset of frailty at follow-up (RR = 1.30, 95% CI: 1.02–1.64). Among specific sleep health dimensions, lack of daytime alertness and short (< 6 hours) or long (≥ 9 hours) sleep duration were significantly associated with frailty, however, poor sleep satisfaction, timing and efficiency were not significantly associated with frailty. Stratified analyses revealed that the association between sleep health and frailty was significant among women, those aged <80 years, and non-Hispanic Whites after adjusting for socio-demographics, but only significant among women (RR = 1.44, 95% CI: 1.04–1.98) after adding health correlates. Findings suggest that having poorer sleep health across multiple dimensions is associated with increased risk of frailty among older adults, especially among women.

